# Oral Psychosomatic Disorders in Family Caregivers of Oral Squamous Cell Carcinoma Patients

**DOI:** 10.31557/APJCP.2021.22.2.477

**Published:** 2021-02

**Authors:** Shailesh M. Gondivkar, Amol R. Gadbail, Sachin C. Sarode, Amol Hedaoo, Subhrajit Dasgupta, Balkrishna Sharma, Apparna Sharma, Monal Yuwanati, Rima S. Gondivkar, Rahul N. Gaikwad, Gargi S Sarode, Shankargouda Patil

**Affiliations:** 1 *Department of Oral Medicine & Radiology, Government Dental College & Hospital, Nagpur Maharashtra, India. *; 2 *Department of Dentistry, Indira Gandhi Government Medical College & Hospital, Nagpur, Maharashtra State, India. *; 3 *Department of Oral Pathology & Microbiology, Dr. D.Y. Patil Dental College & Hospital, Dr. D.Y. Patil Vidyapeeth, Pune, Maharashtra State, India. *; 4 *Rashtra Sant Tukdoji Cancer Hospital & Research Centre (Tertiary care cancer centre), Nagpur, Maharashtra State, India. *; 5 *Department of Dentistry, Government Medical College & Hospital, Nagpur, Maharashtra State, India. *; 6 *Department of Oral Pathology & Microbiology, College of Dental Science & Research Centre, People’s University, Bhopal, Madhya Pradesh, India. *; 7 *Independent Researcher, Aarti regency, Mahalakshmi Nagar, Manewada Road, Nagpur, Maharashtra State, India. *; 8 *Department of Community Dentistry and Oral Epidemiology, College of Dentistry, Qassim University, Buraydah, Kingdom of Saudi Arabia. *; 9 *Department of Maxillofacial Surgery and Diagnostic Sciences, Division of Oral Pathology, College of Dentistry, Jazan University, Jazan, Kingdom of Saudi Arabia. *

**Keywords:** Oral cancer, DASS test, caregivers, oral psychosomatic disorders

## Abstract

**Objective:**

To investigate the oral psychosomatic disorders (PSDs) in family caregivers (FCs) of oral cancer (OC) patients and to evaluate the correlation between these oral PSDs to severity of depression anxiety and stress.

**Methods:**

A total of 50 participants were included each in first degree relative (FDR), second degree relative (SDR) and control group. All the participants completed DASS-21 questionnaire and were subjected to thorough clinical history and oral examination.

**Results:**

All the FCs reported statistically significant higher mean levels of depression, anxiety and stress compared to controls (p˂0.001). A significantly greater number of FCs (40.00%) reported oral PSDs than control group (12.00%). Most prevalent oral PSD in FCs was aphthous stomatitis followed by oral lichen planus, bruxism, burning mouth syndrome and myofascial pain dysfunction syndrome. Moreover, there was a preponderance of these diseases in FDR (60.86%) compared to SDR (26.08%). FCs with moderate to very severe depression, anxiety and stress showed higher prevalence of these oral PSDs compared to the ones with mild depression, anxiety and stress.

**Conclusion:**

The observations of higher prevalence of oral PSDs in FCs with psychological alterations can enhance healthcare professionals’ awareness to better understand FCs’ oral healthcare needs.

## Introduction

Over 1,000,000 new cases of oral squamous cell carcinoma (OSCC) are registered every year in India (Warnakulasuriya, 2009). In India, a high proportion of patients with OSCC are from lower socioeconomic classes. This high proportion is clearly associated with difficulties in accessing the health care system, with most cases eventually diagnosed at advanced clinical stages. Moreover, oral cancer patients also suffer from workday losses and work-related disabilities making both ends meet very difficult. OSCC treatment sequel are related not only to cosmesis but also mainly to the patient’s ability to communicate and to vital functions such as chewing and swallowing. Consequently, this causes potentially devastating socioeconomic effects for the patients and as well as their families (Vartanian et al., 2006). As a result, relatives and friends, commonly known as informal or family caregivers (FCs), often become an indispensable part of a patient’s support team during the treatment and post-treatment period. Many times, FCs need to play their role in sudden circumstances without any preparation and faces multiple challenges. The literature indicates that FCs often report experiencing deficits in psychological health and functioning, which has been defined as encompassing emotional distress and depressive and anxious symptoms (Ross et al., 2010; Hodges and Humphris, 2009; Röing et al., 2008). Since providing care to cancer patients is a consistent role for longer duration, all these problems ultimately results in increasing FCs’ burden (Hwang et al., 2003; Schubart et al., 2008) and deteriorates their quality of life (Romito et al., 2013; Kim and Given, 2008).

A psychosomatic disorder (PSD) involves both body and mind. These diseases results in physical and pathological changes originating from mental or emotional causes. Most common ones are stress, anxiety and depression. Since oral cavity is related directly to the human instincts and passions, a wide spectrum of psychiatric disorders affects oral and para oral structures, which have a definite psychosomatic cause. The psychological alterations and emotions may result in development and worsening of oral mucosal diseases (Soto Araya et al., 2004; Valter et al., 2013). Although, till date, very few researchers focused mainly on understanding the psychosocial impact and assessment of caregiving burden of FCs of oral cancer patients (Goswami et al., 2019; Goswami and Gupta, 2020) but no study has yet explored the prevalence of oral psychosomatic disorders in FCs of oral cancer patients.

With this view in mind, present study was designed to investigate the oral cavity of the FCs for possible presence of oral PSD and correlate it with the degree of depression, anxiety and stress. Emphasis was also given for the time frame as manifestation of oral PSD also depends on duration of psychological distress. 

**Table 1:** Demographic Characteristics of the Participants

Characteristics	FDR n (%)	SDR n (%)	Control n (%)
Total sample	50	50	50
Age			
mean (SD)	38.18 (±4.88)	40.34 (±5.48)	36.16 (±4.34)
Range	28 – 52	30 - 58	28 - 46
Gender			
Male	12 (24.00)	40 (80.00)	36 (72.00)
Female	38 (76.00)	10 (20.00)	13 (26.00)
Marital status			
Married	45 (90.00)	48 (96.00)	46 (92.00)
Unmarried/divorced/ widowed	5 (10.00)	2 (4.00)	4 (8.00)
Living together with the patient			
Yes	46 (92.00)	12 (24.00)	NA
No	4 (8.00)	38 (76.00)	NA
Highest level of education			
Primary	9 (18.00)	11 (22.00)	10 (20.00)
Secondary	29 (58.00)	23 (46.00)	27 (54.00)
College/university	12 (24.00)	16 (32.00)	13 (26.00)
Employment status			
Full time/part-time work	39 (78.00)	38 (76.00)	38 (76.00)
Unemployed	4 (8.00)	6 (12.00)	0 (00.00)
Retired/other	7 (14.00)	6 (12.00)	12 (24.00)

FDR, First degree relative; SDR, Second degree relative; SD, Standard Deviation; NA, Not applicable

**Table 2:** Mean Levels of Depression, Anxiety, Stress and Total DASS Sscore in Various Groups

Parameter	Groups	n	Mean (SD)	P-value
Depression	Control (A)	50	1.54 ±1.99	A<B (p<0.0001);
	FDR (B)	50	9.10 ±4.96	C<B (p<0.0001);
	SDR (C)	50	5.16 ±3.64	A<C (p<0.0001);
	All FCs (D)	100	7.13 ±4.76	A<D (p<0.0001)
	FCs with oral PSDs	40	11.80 ±3.44	p<0.0001
	FCs without oral PSDs	60	4.01 ±2.36	
Anxiety	Control (A)	50	1.20 ±1.67	A<B (p<0.0001);
	FDR (B)	50	7.40 ±4.41	C<B (p<0.0001);
	SDR (C)	50	4.34 ±2.97	A<C (p<0.0001);
	All FCs (D)	100	5.87 ±4.04	A<D (p<0.0001)
	FCs with oral PSDs	40	9.57 ±3.56	p<0.0001
	FCs without oral PSDs	60	3.40 ±1.8	
Stress	Control (A)	50	1.86 ±2.88	A<B (p<0.0001);
	FDR (B)	50	11.92 ±4.96	C<B (p<0.0001);
	SDR (C)	50	7.56 ±4.42	A<C (p<0.0001);
	All FCs (D)	100	9.74 ±5.16	A<D (p<0.0001)
	FCs with oral PSDs	40	14.60 ±3.24	p<0.0001
	FCs without oral PSDs	60	6.50 ±3.34	
Total DASS score	Control (A)	50	4.60 ±6.36	A<B (p<0.0001);
	FDR (B)	50	28.42 ±14.10	C<B (p<0.0001);
	SDR (C)	50	17.06 ±10.93	A<C (p<0.0001);
	All FCs (D)	100	22.74 ±13.79	A<D (p<0.0001)
	FCs with oral PSDs	40	35.97 ±10.04	p<0.0001
	FCs without oral PSDs	60	13.91 ±7.37	

FDR, First degree relative; SDR, Second degree relatives; FCs, Family caregivers; PSDs, Psychosomatic disorders; SD, Standard Deviation.

**Table 3.** Comparison of Scores of Psychological Parameters between the Control Groups, First-Degree Relatives and Second-Degree Relatives

Parameter	Scale	FDR n (%)	SDR n (%)	Control n (%)	Total n (%)	P value
Depression, Anxiety and Stress	Normal	9 (18.00)	18 (36.00)	43 (86.00)	70 (46.66)	p<0.0001
	Mild	14 (28.00)	19 (38.00)	6 (12.00)	39 (26.00)	
	Moderate	8 (16.00)	7 (14.00)	1 (2.00)	16 (10.66)	
	Severe	11 (22.00	4 (8.00)	0 (0.00)	15 (10.00)	
	Very severe	8 (16.00)	2 (4.00)	0 (0.00)	10 (6.66)	
	Total	50	50	50	150	

FDR, First degree relative; SDR, Second degree relative

**Table 4.** Comparison of Scores of Psychological Parameters between the Control Groups, Caregivers with Psychosomatic Disorders and without Disorders

Parameter	Scale	FCs with oral PSDs n (%)	FCs without oral PSDs n (%)	Control n (%)	Total n (%)	P value
Depression, Anxiety and Stress	Normal	0 (0.00)	27 (45.00)	43 (86.00)	70 (46.66)	p<0.0001
	Mild	0 (0.00)	33 (55.00)	6 (12.00)	39 (26.00)	
	Moderate	15 (37.50)	0 (0.00)	1 (2.00)	16 (10.66)	
	Severe	15 (37.50)	0 (0.00)	0 (0.00)	15 (10.00)	
	Very severe	10 (25.00)	0 (0.00)	0 (0.00)	10 (6.66)	
	Total	40	60	50	150	

FCs, Family caregivers; PSDs, psychosomatic disorders

**Table 5:** Distribution of Oral Psychosomatic Ddisorders in Family Caregivers and Control Groups

Characteristics	FDR n (%)	SDR n (%)	Control n (%)	Total n (%)	Chi-square test
Aphthous stomatitis	15 (60.00)	6 (24.00)	4 (16.00)	25 (54.34)	
Oral lichen planus	6 (54.54)	3 (27.27)	2 (18.18)	11 (23.91)	P = 0.003
Bruxism	3 (60.00)	2 (40.00)	0 (00.00)	05 (10.86)	
Burning mouth syndrome	2 (66.66)	1 (33.33)	0 (00.00)	03 (6.52)	
Myofascial pain dysfunction syndrome	2 (100.00)	0 (00.00)	0 (00.00)	02 (4.34)	
Total	28 (60.86)	12 (26.08)	06 (13.04)	46	

FDR, First degree relative; SDR, Second degree relative

## Materials and Methods

*Study design*

A cross-sectional study was conducted on FCs of OSCC patients. 

*Subjects*

To be eligible for the study, potential participants had to (a) have a family member with oral cancer, (b) be the identified or self-identified family FCs for that family member, (c) be at least 18 years of age, (d) not have a history of prior or current psychiatric or neuropsychological disorders and/or not undergoing treatment for any psychosomatic disorder and (e) not have a diagnosis of cancer of any type. Participants had to be able to complete the questions themselves, although an assistant could act as an amanuensis. All participants provided their informed consent on receiving a full explanation of the purpose of the study, and there were no financial implications for the subjects. The family FCs participated in the study were categorized into first degree relatives (FDR- close blood relative which includes individual’s parents, full siblings, children and spouse) and second-degree relative (SDR- blood relative which includes the individual’s grandparents, grandchildren, aunts, uncles, nephews, nieces or half-siblings). A total of 100 FCs were included in the present study (FDR:50; SDR:50). The controls were recruited from the relatives of those patients visited for routine check of oral hygiene/dentition in outpatient department after obtaining informed consent. The total 50 age and sex matched healthy individuals were included as controls. 

*Questionnaire*

The present study employed DASS-21 questionnaire that consists of 21 items divided into three subscales of seven items each to evaluate depression, anxiety, and stress respectively (Lovibond and Lovibond, 1995). 

*Procedure*

All the participated FCs and control subjects were provided DASS-21 questionnaire and asked to rate the extent to which they had experienced each symptom over the previous week on a four-point scale ranging between 0 (“did not apply to me at all”) and 3 (“applied to me very much or most of the time”). Subsequently, the total number of points for each subscale was calculated and multiplied by two to calculate the final score. Based on the score obtained, the extent of a given negative emotion was classified as normal, mild, moderate, severe, or extremely severe (Lovibond and Lovibond, 1995). A thorough clinical examination of the oral cavity of all the participants was carried out under artificial illumination on a dental chair for identification of any PSD. Selected patients were then investigated for diagnosis using appropriate diagnostic workups. 

BMS was recognized when oral burning or pain was present in the absence of detectable mucosal changes at the time of clinical examination. The diagnosis of apthous ulceration was based on patients history and clinical findings. Patients reporting with history of recurrent episodes of round or ovoid ulcers surrounded by erythematous halo and each episode of ulceration lasting for a few days to weeks (Suresh et al., 2014).

The clinical diagnosis of oral lichen planus was established as described by Suresh et al., 2014. Laskin’s diagnostic criteria was applied for evaluation of myofascial pain dysfunction syndrome (Nirupama et al., 2018) and bruxism was diagnosed according to the recommendations from the third edition of the International Classification of Sleep Disorders (ICSD-3) published by the American Academy of Sleep Medicine (AASM) (Sateria MJ 2014).

*Statistical analysis*

The data was analyzed statistically using Statistical Package for the Social Sciences, version 17.0. One-way ANOVA and Tukey HSD test were applied to find out the differences in mean score of anxiety, stress and depression amongst the groups. The comparison of mean score of anxiety, stress and depression between individual groups were carried out by student t test. The chi-square test was used to compare the oral PSDs with different level of anxiety, stress and depression and in FCs and control group. The level of statistical significance was at p<0.05.

## Results

*Demographic characteristics*

A total of 100 FCs (related to 100 OSCC patients) and 50 controls were enrolled in the present study. Amongst 100 FCs, 50 were categorized as FDR and SDR each. Out of 50 FDR, 38 were females and 12 were males whereas, SDR included 40 males and 10 females. The age of the FDR ranges from 28 to 52 years with mean of 38.18 (±4.88) while SDR were in age range of 30 and 58 with mean of 40.34 (±5.48). The majority of the FCs were married (93%) and 58% were living together with the patient. More than half of the FCs had secondary school school education and majority (77%) had full- or part-time employment. The control group included 36 males and 13 females with mean age of 36.16 (±4.34). (Table 1).

*DASS test and FCs*

The FCs exhibited significantly higher mean levels of depression, anxiety, and stress compared with the control subjects (p<0.001). Similarly, FDR demonstrated significantly higher mean levels of depression, anxiety, and stress than SDR (p<0.001) (Table 2). 

Severe (10.00%) and very severe (6.66%) scores of depression, anxiety and stress were reported in the FCs group only. The majority of control subjects (86.00%) exhibited a normal level of depression. Higher percentage of moderate (16.00%), severe (22.00%) and very severe (16.00%) scores of depression, anxiety and stress were reported in FDR as compared to moderate (14.00%), severe (8.00%) and very severe (4.00%) scores of depression, anxiety and stress in SDR (Table 3).

*DASS test and oral PSDs*

The FCs with oral PSDs exhibited significantly higher mean levels of depression, anxiety, and stress compared with the FCs without any PSD (p<0.001) (Table 2). All the FCs with PSDs were reported with 37.50% moderate, 37.50% severe, and 25.00% very severe scores of depression, anxiety and stress. Whereas, all FCs without any PSDs were reported with 45.00% normal and 55.00% mild levels of depression, anxiety and stress (Table 4). 

*Oral psychosomatic disorders*

Out of total 100 FCs, forty were affected with oral PSDs and only 6 amongst control group had oral PSDs. In FCs group, most common oral PSD reported was aphthous stomatitis (AS), followed by oral lichen planus (OLP), bruxism and burning mouth syndrome (BMS). In addition, myofascial pain dysfunction syndrome (MPDS) was reported in only two patients. It was observed that FDR showed more oral PSDs as compared to SDR. In FDR, the most common condition found was AS followed by OLP, bruxism and burning mouth syndrome. SDR also showed similar trend for PSDs. Both the reported cases of MPDS belonged to FDR group. In the control group, 4 participants showed minor AS and reticular OLP was recorded in two (Table 5).

FCs in the fourth and fifth decades were most commonly affected with AS (27.50% & 22.70%), followed by OLP (24.60% & 28.30%) and bruxism (23.10% & 22.50%) respectively. Although, BMS was reported more in fifth and sixth decades (28.70% & 26.70%), both the cases of MPDS were in the age group of fifth decade. In control group, 3 participants with AS and one participant with OLP were in fourth decade and remaining noticed to be in fifth decade. When distribution among the gender in 40 FCs with oral PSDs was considered, we observed more prevalence of AS (n=13), OLP (n=6) and BMS (n=2) in females while greater number of bruxism (n=4) and MPDS (n=2) cases were reported in males. 

Minor AS was the most common form noticed and was present in 19 FCs (90.47%), whereas only 2 (9.52%) FCs had major AS. The buccal mucosa (63.15%) and labial mucosa (26.31%) were the commonest sites of involvement of AS. Out of 19 FCs with minor AS, 6 (31.57%) showed ulcers involving right buccal mucosa, 3 FCs (15.78%) each reported ulcers on left buccal mucosa and bilateral buccal mucosa involvement, lower and upper labial mucosa was involved in 3 (15.78%)) and 2 (10.52%) FCs respectively and remaining 2 (10.52%) FCs had multifocal involvement in oral cavity. 

Amongst 9 OLP cases, five FCs (55.55%) showed reticular form, three (33.33%) showed erosive type and only one had plaque form of OLP. Bilateral involvement of buccal mucosa was reported in 6 FCs (66.66%) with OLP, followed by tongue (22.22%) and one FC (11.11%) had lesions on gingiva. Out of 6 FCs of OLP with bilaterally involved buccal mucosa, 2 cases (33.33%) showed involvement in other oral sites including tongue and gingiva. Out of 5 FCs with reticular OLP, 3 showed bilateral buccal mucosa involvement, 2 had left buccal mucosa involvement and one had lesions on bilateral buccal mucosa with collateral tongue involvement. Similarly, out of 3 FCs with erosive OLP, bilateral buccal mucosa involvement was reported in 2 FCs and bilateral buccal mucosa with collateral gingiva was seen in one FC. The only FC with plaque form of OLP showed lesions on buccal mucosa bilaterally. 

Out of total 5 cases of bruxism, four were noticed in males in contrast to one female FC. The reported patterns of bruxism were diurnal (80.00%) and nocturnal (20.00%). Two (40.00%) of the 5 bruxists FCs showed both tooth mobility and chewing difficulty while other two (40.00%) reported teeth sensitivity and one (20.00%) reported temporomandibular joint pain/noise. 

Two of the 3 FCs with BMS were females. The tongue (n=3) was the most commonly affected anatomic location in conjunction with other sites including buccal mucosa (n=2), lips (n=2), palate (n=1) and gingiva (n=1). All the three FCs werer reported mild to moderate pain. One FC reported oral dryness and slightly altered taste. The present study noted only 2 FCs with MPDS and both were males having deep bite. The most common muscular involvement was related to lateral pterygoid muscle (n=2) in conjunction with temporalis (n=1) and medial pterygoid (n=1) muscles. Both the FCs showed slight limitation in mouth opening, jaw deviation and pain in the masticatory muscles along with headache and neck pain. 

## Discussion

In contrast to previous research on FCs, this study to the best of our knowledge is the first of its kind to evaluate and correlate the oral PSDs and psychological parameters in FCs of OSCC patients. The most notable finding was the higher prevalence of oral PSDs and higher levels of depression, anxiety and stress in FCs of OSCC patients than in general population of similar age. Since FDR are more closely associated with the patient, they demonstrated significantly higher scores of depression, anxiety, and stress compared with the SDR. Looking at the continuously increasing number of OSCC cases globally, there was a dire need of investigating the oral PSDs in FCs of these patients so that management of their healthcare needs should be taken into consideration.

All the FCs reported significantly higher mean levels of depression, anxiety and stress as compared to control group (p˂0.001). This is consistent with the previous research which demonstrated that caregivers of cancer patients suffer from depression, anxiety and stress (Blanchard et al., 1997; Rigoni et al., 2016; Northouse et al., 2012). Once a person is diagnosed with cancer, his /her family member goes through complex emotional mix-ups due to deep-seated fear of losing their near and dear ones. As majority of the Indian population are living in joint family, each member play a specific role in taking care of cancer patient and therefore face a different degree of impacts. However, those, who are directly involved in providing care, are more vulnerable to suffer from emotional stress. In the present study, greater number of FCs in FDR group reported severe and very severe scores of depression, anxiety and stress than in SDR group. Similarly, Northouse and Peters-Golden in their study, reported depression in 22.7% FCs and 2.7% had severe depression (Northouse and Peters-Golden, 1993).

In the present study, significantly greater numbers of FCs (40.00%) were affected with oral PSDs than control group (12.00%). Moreover, there was a preponderance of oral PSDs in FDR (60.86%) of OSCC patients as compared to SDR (26.08%). This goes in accordance with the high degree of depression, anxiety and stress in FDR. The most common oral PSDs reported in FCs were AS followed by OLP, bruxism, BMS and MPDS. These results are consistent with the prevalence of these diseases in population with anxiety, depression wherein AS, OLP and BMS were the most common PSDs affecting oral mucosa (Suresh et al., 2014). As oral mucosa is extremely complex, delicate and highly responsive to emotional status of the individuals, numerous researchers tried to correlate exacerbation of oral mucosal diseases like AS, OLP and BMS with emotional instability, psychological state and personality changes but few evaluated the prevalence of oral mucosal diseases in anxiety, depression and other psychiatric disorders (Suresh et al., 2014; Dangore-Khasbage et al., 2012). The prevalence of AS, bruxism and BMS found in this study is consistent with the past studies conducted in general population (Patil et al., 2014; Seligman et al., 1988; Kohorst et al., 2014). However, in line with a few other studies, we found higher prevalence rate of OLP and lower prevalence of MPDS (Murti et al., 1986; Mortazavi et al., 2010). The higher prevalence of OLP in our study may be attributed to severe and very severe scores of depression, anxiety and stress especially in FDR group. In this study, the mean age of FCs was 39.23 (±3.48). At this age decade, prevalence of MPDS is as such lower as compared to other oral PSD such as AS, LP and BMS. Hence, very few cases of MPDS were reported in the present study. 

It has been advocated that psychological strain procreate the initiation and exacerbation of oral mucosal diseases. Psychological alterations establish its impact on body by the multidirectional and close interrelations among the nervous, immune and endocrine system (Schiavone et al., 2012). The present study demonstrated significantly higher scores of depression, anxiety, and stress in FCs with oral PSDs than FCs without any oral PSD. Although no known study has yet evaluated oral PSDs in FCs of OSCC patients, few researchers studied stress, anxiety and depression levels in general as well as defined population with oral mucosal diseases. They reported significantly higher stress, depression and anxiety levels in AS, OLP and BMS patients than control groups (Valter et al., 2013; Schiavone et al., 2012; Gallo et al., 2009).

Restricted study participants is the major limitation of the present study. Looking at the continuously increasing prevalence of OSCC, particularly in India, a nationwide multicentric study is warranted to achieve better insights in regard to oral PSDs in FCs of these patients. The present study did not considered socioeconomic status of the participants which might have important role in increasing the burden of FCs and subsequently leading to oral PSDs.. Moreover, estimation of biological markers in addition to stress, anxiety and depression scores would have added value to the accurate assessment of psychological alterations in study participants. 

One main contribution of this cross-sectional study is that it extends previous research and helps to fill the gap in knowledge about the presence of oral PSDs and their correlation with depression, anxiety and stress in FCs of OSCC patients. The noteworthy finding in our study was the higher prevalence of AS, OLP, bruxism, BMS and MPDS in FCs than healthy individuals with sound mind. The moderate to very severe scores of depression, anxiety and stress in FCs with oral PSDs showed a strong positive association. We recommend future prospective studies with larger sample size that can help enhance healthcare professionals’ awareness to better understand and identify FCs’ oral healthcare needs in the initial phases of the illness so that early interventions can be provided to halt the evolution or minimize the symptoms of the diseases. 

## Declaration

The study protocol was approved by the institutional ethics committee of Cancer Relief Society’s RST Regional Cancer Hospital, Nagpur India, in June 2019 (approval no. RST/RCH: 444).

## Note

This study was carried out at Rashtra Sant Tukdoji Cancer Hospital & Research Centre (Tertiary care cancer centre), Nagpur, Maharashtra State, India. The study protocol was approved by the institutional ethics committee of Cancer Relief Society’s RST Regional Cancer Hospital, Nagpur India, in June 2019 (approval no. RST/RCH: 444).
